# Olanzapine add-on treatment promotes neuronal differentiation of neural stem cells compared with fluoxetine alone

**DOI:** 10.1042/BSR20220804

**Published:** 2022-10-18

**Authors:** Jiantong Sun, Danlian Wu, Guangjuan Xu, Fang Chen, Xinyuan Ding, Linjun Xie, Zhangfeng Yu, Xing Jin

**Affiliations:** 1 Department of Pharmacy, The Affiliated Suzhou Hospital of Nanjing Medical University, Baita West Road (16#), Suzhou, China; 2 Department of Pharmacy, The Affiliated Jiangyin Clinical College of Xuzhou Medical University, Shoushan Road (163#), Jiangyin, China; 3 Gusu School, Nanjing Medical University, Shizi Street (458#), Suzhou, China; 4 Department of Critical Care Medicine, The Affiliated Suzhou Hospital of Nanjing Medical University, Baita West Road (16#), Suzhou, China; 5 Suzhou Clinical Medical Center of Critical Care Medicine, Suzhou, China

**Keywords:** fluoxetine, neural stem cells, neurogenesis, neuronal differentiation, olanzapine

## Abstract

The addition of olanzapine to fluoxetine produces an antidepressant effect on fluoxetine nonresponders. Promoting hippocampal neurogenesis is associated with the successful treatment of depression. The present study aimed to investigate the interaction of olanzapine and fluoxetine in regulating neurogenesis. We found that fluoxetine alone does not affect cell proliferation and inhibits the neuronal differentiation of cultured neural stem cells (NSCs), but promotes NSCs proliferation and exerts no effect on neuronal fate when NSCs are cocultured with neurons. In addition, fluoxetine alone also does not alter the neuronal fate of newborn hippocampal cells *in vivo*. Although fluoxetine treatment elicits different results, our data consistently show that olanzapine alone does not affect the proliferation and neuronal differentiation of NSCs. The combination of olanzapine and fluoxetine has no profound effect on NSCs proliferation compared with fluoxetine alone, but olanzapine add-on treatment produces a greater number and percentage of differentiated neurons from NSCs. Further investigations are needed to explore the underlying mechanisms of the increased neurogenesis caused by the combination of olanzapine with fluoxetine.

## Introduction

Depression is a highly prevalent psychiatric disease and a leading cause of disability worldwide [1]. Antidepressant medications are commonly used to treat various forms of depression; however, a large number of patients fail to respond adequately to antidepressant monotherapies. Low-dose atypical antipsychotics such as olanzapine are effective for augmenting the action of antidepressants. The fixed-dose oral formulation of fluoxetine and olanzapine has been approved by the US Food and Drug Administration for treatment-resistant depression and acute-depressive episodes associated with bipolar І disorder [2]. Despite the frequent use in clinical practice, the mechanisms by which olanzapine augments the antidepressant effect of fluoxetine are poorly understood.

Neurogenesis in the hippocampus is known to be associated with depression and antidepressant effects. Active neurogenesis, a process of generating new neurons from neural stem cells (NSCs), persists throughout the adult life of mammals including humans in restricted brain regions, particularly in the dentate gyrus (DG) of the hippocampus, a brain structure that is crucial for regulating emotion [3,4]. In middle-aged humans, 700 new neurons are added in each hippocampus per day [5]. Studies demonstrate that the volume of DG is decreased in depressed patients and hippocampal neurogenesis is impaired in animal chronic stress models [6,7]. Fluoxetine reverses the suppression of hippocampal neurogenesis caused by stress, and disrupting hippocampal neurogenesis blocks behavioral responses to fluoxetine [8,9]. Furthermore, in fluoxetine-resistant depressive rats, decreased hippocampal neurogenesis coexists with poor behavioral results [10]. Thus, promoting hippocampal neurogenesis seems to be a key step in the successful treatment of depression. So far, we do not know whether the underlying mechanism of augmenting the antidepressant effect of fluoxetine with olanzapine may be explained by increased hippocampal neurogenesis.

In this study, we aimed to investigate whether olanzapine can augment the effects of fluoxetine on NSCs proliferation and neuronal differentiation by cell culture *in vitro* and animal experiments. Our data demonstrate that the combination of olanzapine and fluoxetine does not further enhance proliferation of NSCs compared with fluoxetine alone, but olanzapine add-on treatment produces a greater number and percentage of differentiated neurons from NSCs.

## Materials and methods

### Animals

Adult male (8–10 weeks old) and embryonic ICR mice purchased from Shanghai Silaike Laboratory Animal (Shanghai, China) were used in the present study. Mice were housed under a 12-h light/12-h dark cycle and in a temperature- and humidity-controlled environment. All experimental protocols using animals were approved by the Institutional Animal Care and Use Committee of Nanjing Medical University.

### Drugs

Fluoxetine was purchased from Sigma-Aldrich (St. Louis, MO, USA). Olanzapine was provided by the manufacturer (Hansoh Pharma, Lianyungang, China). For cell experiments, fluoxetine and olanzapine were, respectively, dissolved in sterilized water and dimethylsulfoxide (DMSO). For animal experiments, fluoxetine was dissolved in saline; olanzapine was dissolved in 1 M HCl in water and then the pH was adjusted to 6.0 with 1 M NaOH.

### Cell cultures

NSCs were isolated from embryonic day 14 (E14) mouse cortex as we previously described [11]. Cells were floating cultured in proliferation medium, DMEM/F12 medium (1:1; Invitrogen) containing 20 ng/ml basic fibroblast growth factor (bFGF; Sigma-Aldrich), 20 ng/ml epidermal growth factor (EGF; Sigma-Aldrich), and 2% B27 supplement (Invitrogen), and passaged every 4–6 days. Embryonic NSCs of the second to fifth passage were used in the present study.

Primary neurons were isolated from E16 mouse cortex. These cortices were cut into pieces and then were digested with 0.125% trypsin (Invitrogen) at 37°C for 10 min. DMEM/F12 medium containing 10% fetal bovine serum (FBS; TBD) was used to terminate the digestion. Dissociated cells were filtered through a cell strainer with 200 screen mesh. Finally, the single-cell suspension was planted into dishes coated with polyornithine (10 μg/ml; Sigma-Aldrich) at 1 × 10^5^ cells/cm^2^ in Neurobasal medium (Invitrogen) containing 2% B27 supplement.

In the coculture of NSCs with neurons, single-cell suspension of NSCs was planted on coverslips coated with polyornithine (10 μg/ml) and laminin (5 μg/ml; Invitrogen) at 2 × 10^4^ cells/cm^2^ and allowed to proliferate for 24 h. Then, the coverslips with NSCs were turned over and put on the primary neurons, which had been cultured in dishes for 10 days. Proliferation medium (Neurobasal medium containing 20 ng/ml bFGF, 20 ng/ml EGF, and 2% B27) or differentiation medium (Neurobasal medium containing 2% B27 and 0.8% FBS) was used as cocultured medium. The coverslips with NSCs were removed from the dishes to perform the immunocytochemistry of NSCs after the coculture.

All cultures, including NSCs, neurons, and cocultures, were maintained in an incubator (HERAcell 150, Thermo Fisher Scientific) with humidified atmosphere of 95% air and 5% CO_2_ at 37°C.

### NSCs proliferation assays

BrdU incorporation and cell counting experiments were used to assess cell proliferation. For BrdU incorporation, single-cell suspension of NSCs was seeded on polyornithine/laminin-coated coverslips at 2 × 10^4^ cells/cm^2^, cultured as a monolayer. Drugs (fluoxetine and/or olanzapine) were added when monolayer-cultured NSCs proliferated for 24 h or when cocultures began. Cells were treated with the drugs for 24 h and 10 μM BrdU during the last 2 h of drug treatments. Then, the NSCs on coverslips were fixed in phosphate-buffered solution (PBS) containing 4% paraformaldehyde for 15 min at room temperature. The BrdU^+^ cells were visualized by immunofluorescence label.

For cell counting, single-cell suspension of NSCs was seeded in 12-well plates at 2 × 10^4^ cells/ml and incubated with the drugs when seeding. Three days later, generated neurospheres were dissociated to single cells and the number of cells was counted on a hemocytometer. Data were normalized to the percentage of control.

### NSCs differentiation

NSCs were planted on polyornithine/laminin-coated coverslips at 2 × 10^4^ cells/cm^2^ in NSCs proliferation medium first. Monolayer-cultured NSCs were then allowed to differentiate in the presence of drugs for 4 days when they had proliferated for 24 h or when cocultures began. DMEM/F12 medium containing 2% B27 and 0.5% FBS was used for the differentiation of only cultured NSCs. NSCs on coverslips were fixed and stained with β-III-Tubulin at day 4 after differentiation.

### Immunocytochemistry

Cultured cells were processed for immunofluorescence as described previously [11]. The antibodies used were as follows: mouse anti-β-III-Tubulin (1:200; Millipore Bioscience Research Reagents), mouse anti-BrdU (1:1000; Millipore Bioscience Research Reagents), goat antimouse DyLight 488 (1:400; Jackson ImmunoReasch), and goat antimouse Cy3 (1:200; Jackson ImmunoReasch). Finally, Hoechst 33258 (Sigma-Aldrich) was used to label the nuclei. Positive cells were quantified in at least ten fields systematically across the coverslips from three independent experiments of parallel cultures. The average was regarded as the final value of one sample.

### Immunohistochemistry

After transcardial perfusion with saline followed by 4% paraformaldehyde (PFA, dissolved in PBS), the brains of mice were removed and postfixed overnight at 4°C in 4% PFA. A series of 40 μm coronal sections throughout hippocampus were cut with a freezing microtome. Free-floating sections were treated with 50% formamide/2× standard saline citrate for 2 h at 65°C, washed with PBS, and incubated in 2 mol/l HCl for 1 h at 37°C and then in 0.1 mol/l boric acid (pH 8.5) for 10 min. After washing with PBS three times, the sections were blocked in PBS containing 3% goat serum, 0.1% bovine serum albumin, and 0.3% Triton X-100 (named as blocking buffer) for 1 h at room temperature. Then, the sections were successively incubated with primary antibodies at 4°C overnight and, after thorough washing in PBS, with secondary antibodies for 2 h at room temperature. The used antibodies were diluted in blocking buffer and were as follows: mouse anti-NeuN (1:500; Millipore Bioscience Research Reagents), rat anti-BrdU (1:200; Accurate Chemical & Scientific Corporation), goat antimouse DyLight 488 (1:400; Jackson ImmunoReasch), and goat antirat Cy3 (1:200; Jackson ImmunoReasch). Fluorescent images were captured by using a confocal microscope (LSM700, Zeiss). Every sixth section in all of the 40 μm sections was processed for immunohistochemistry and counting. The number of positive cells in selected sections of each dentate gyrus was summed. Multiplying the sum by 6 was regarded as the final value of one sample.

### Statistical analysis

Comparisons were made with one-way ANOVA followed by Tukey’s *post hoc* test. Data were presented as mean ± SEM. Differences were considered significant when *P*<0.05.

## Results

### Olanzapine does not influence proliferation and neuronal differentiation of cultured NSCs

**Figure 1:**
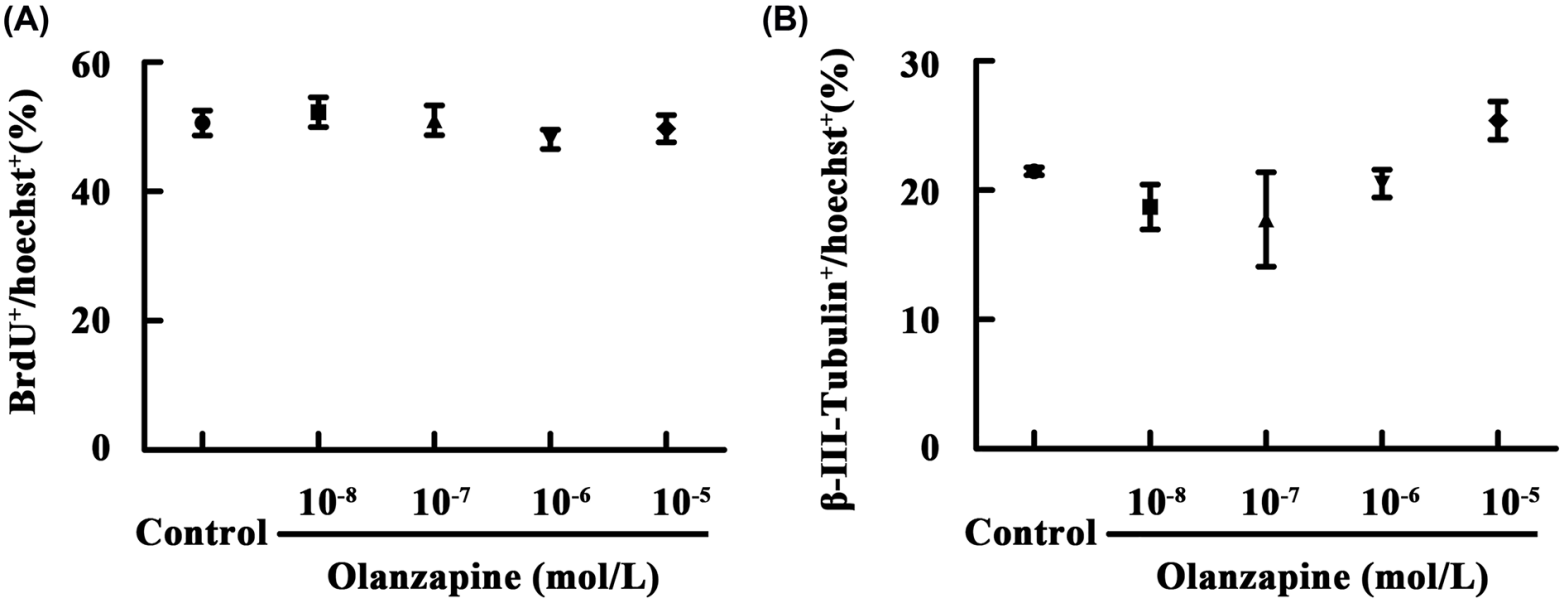
Olanzapine does not influence proliferation and neuronal differentiation of cultured NSCs (**A**) Statistical graph showing the ratio of BrdU-labeled cells of the monolayer-cultured NSCs treated with different concentrations of olanzapine for 24 h. (**B**) Statistical graph showing the ratio of β-III-Tubulin^+^ neurons at day 4 after NSCs differentiation. Cultured NSCs were allowed to differentiate in the presence of different concentrations of olanzapine for 4 days. Data are mean ± SEM (*n*=3). Abbreviations: BrdU, bromodeoxyuridine.

To investigate the role of olanzapine in NSCs proliferation, different concentrations of olanzapine (10 nM, 100 nM, 1 μM, or 10 μM) were added to monolayer-cultured NSCs for 24 h. During the last 2 h of olanzapine treatment, 10 μM BrdU was introduced to label the dividing cells. No remarkable difference in the ratio of BrdU^+^ cells was found between the control and olanzapine-exposed cultures over the concentration range of 10 nM–10 μM (Figure 1A), which indicates that olanzapine does not influence the proliferation of cultured NSCs. Next, we investigated the role of olanzapine in regulating neuronal differentiation. Monolayer-cultured NSCs that had proliferated for 24 h were allowed to differentiate and were simultaneously treated with olanzapine (10 nM, 100 nM, 1 μM, or 10 μM) for 4 days during differentiation. Similarly, there was no significant difference in the percentage of β-III-Tubulin-labeled neurons at day 4 after differentiation across different olanzapine concentrations (Figure 1B). Therefore, olanzapine also had no detectable effect on the differentiation of cultured NSCs into neurons.

### Olanzapine reverses fluoxetine-induced reduction in neuronal differentiation of cultured NSCs

**Figure 2:**
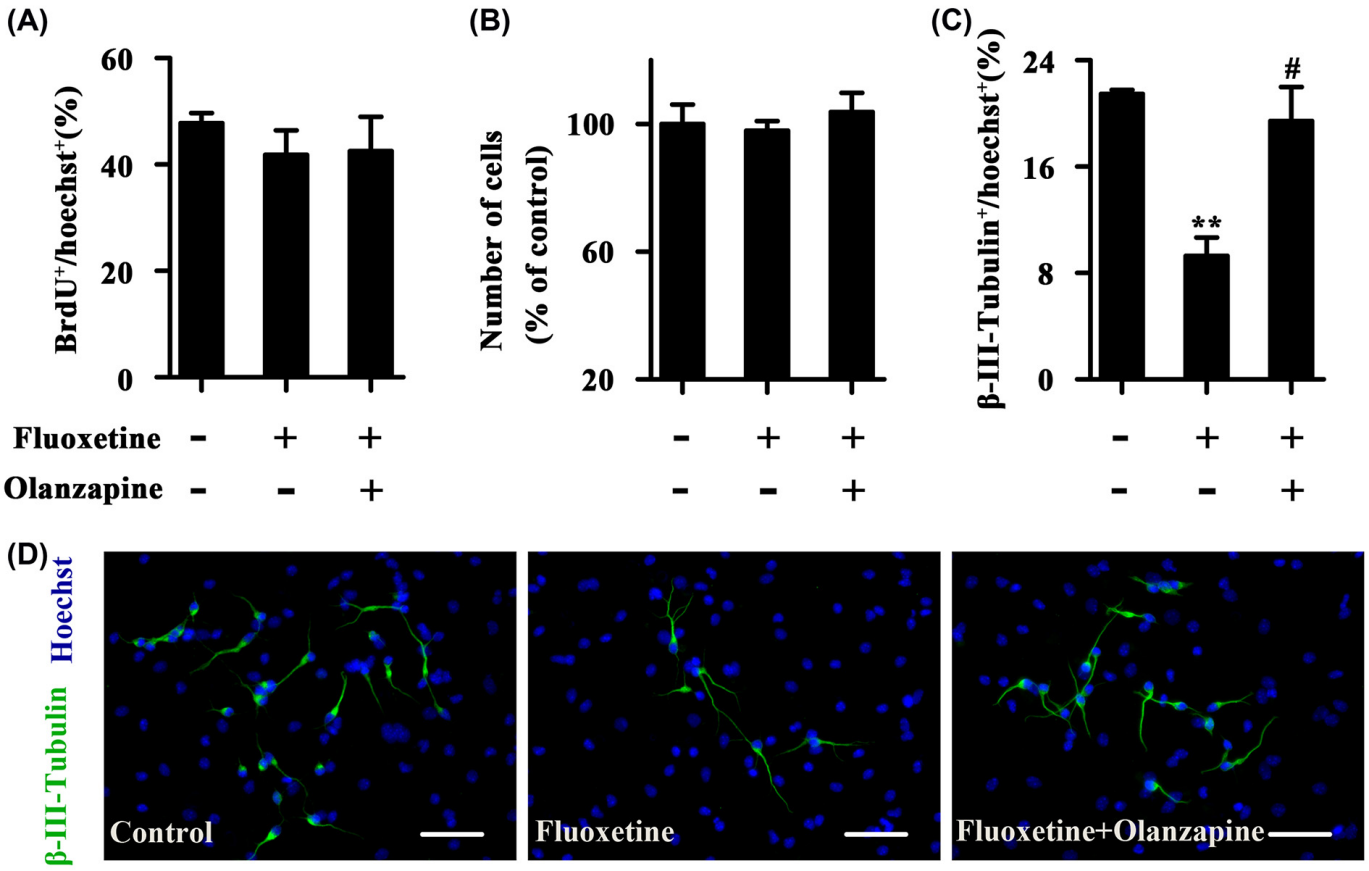
Olanzapine reverses fluoxetine-induced decrease in neuronal differentiation of cultured NSCs (**A**) Statistical graph showing the ratio of BrdU-labeled cells of the monolayer-cultured NSCs treated with fluoxetine or fluoxetine plus olanzapine for 24 h. (**B**) Statistical graph showing the number of cells of neurospheres treated with fluoxetine or fluoxetine plus olanzapine for 3 days. (**C**) Statistical graph showing the ratio of β-III-Tubulin^+^ neurons at day 4 after NSCs differentiation. NSCs were allowed to differentiate in the presence of fluoxetine or fluoxetine plus olanzapine for 4 days. (**D**) Representatives of β-III-Tubulin-labeled cells. Scale bars = 50 μm. Data are mean ± SEM (*n*=3); ** *P*<0.01 versus Control group, # *P*<0.05 versus Fluoxetine group. Abbreviations: BrdU, bromodeoxyuridine.

To examine the interaction between olanzapine and fluoxetine in regulating neurogenesis, we incubated cultured NSCs with 10 μM fluoxetine in the absence or presence of 10 nM olanzapine. The effect of drug treatments on NSCs proliferation is shown in Figure 2A,B. BrdU incorporation experiments revealed that treatment with fluoxetine alone did not influence the proliferation of monolayer-cultured NSCs, and the effect of fluoxetine also did not differ from that of fluoxetine plus olanzapine (Figure 2A). Moreover, similar results were obtained with cell-counting experiments using neurosphere-cultured NSCs (Figure 2B). These findings suggest that there is no interaction between fluoxetine and olanzapine in promoting NSCs proliferation. The effect of fluoxetine, as well as fluoxetine plus olanzapine, on neuronal differentiation is shown in Figure 2C,D (ratio of β-III-Tubulin^+^ neurons; Control, 21.5 ± 0.3%; Fluoxetine, 9.3 ± 1.4%; Fluoxetine plus olanzapine, 19.4 ± 2.6%; F_(2,6)_ = 14.949, *P*=0.005). Monolayer NSCs were allowed to differentiate in the presence of the drugs for 4 days. Unexpectedly, fluoxetine alone resulted in a marked decrease in the percentage of differentiated neurons (β-III-Tubulin^+^) at day 4 after differentiation (*P*<0.01), and the inhibitory effect of fluoxetine was reversed by olanzapine (*P*<0.05). Therefore, potential interaction exists between fluoxetine and olanzapine in regulating NSCs differentiation into neurons.

### Olanzapine has synergism with fluoxetine in promoting neuronal differentiation of neuron-cocultured NSCs

**Figure 3:**
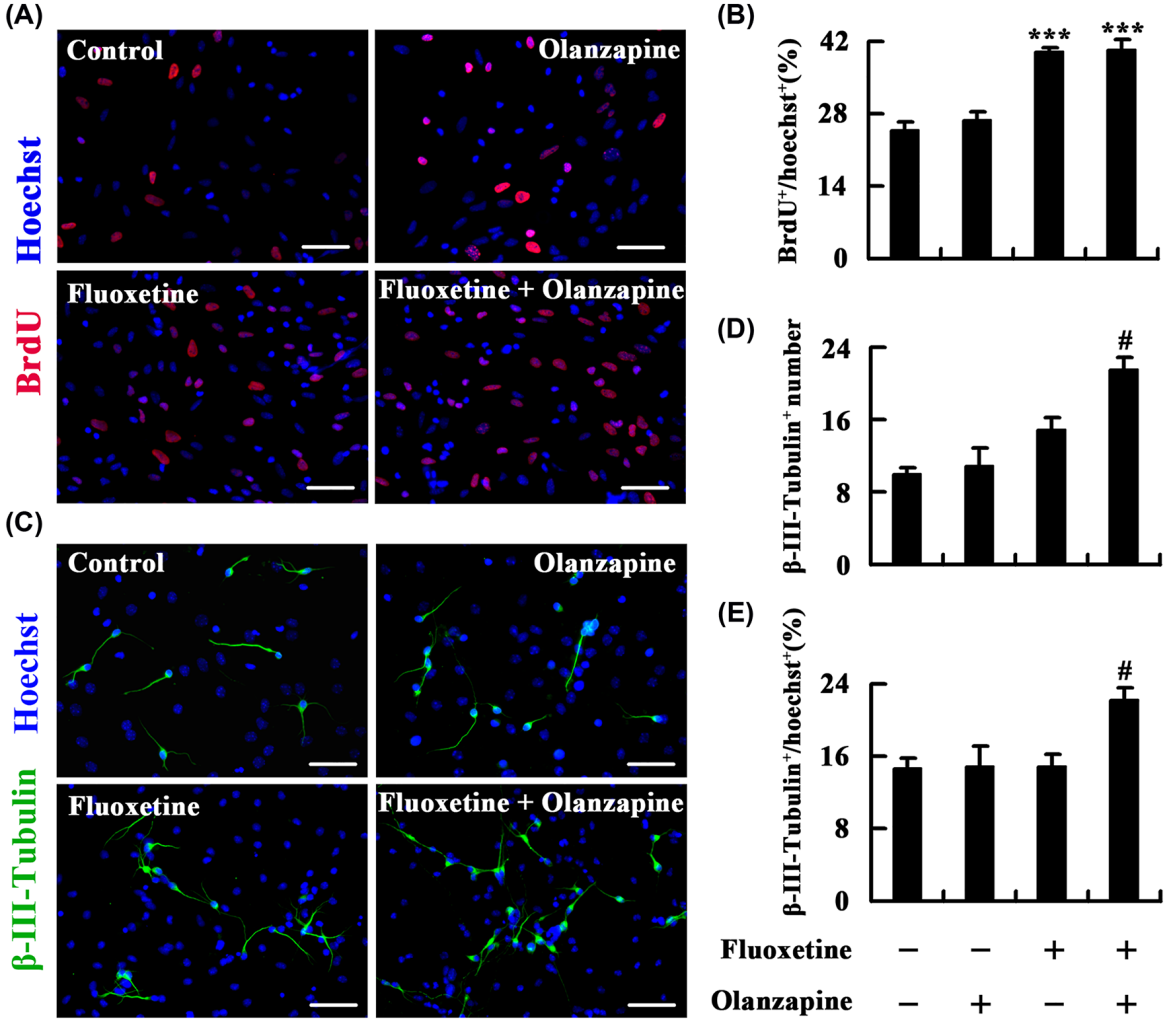
Olanzapine has synergism with fluoxetine in promoting neuronal differentiation of neuron-cocultured NSCs (**A,B**) Effect of fluoxetine and/or olanzapine on proliferation of neuron-cocultured NSCs. Neuron-cocultured NSCs were treated with 10 μM fluoxetine and/or 10 nM olanzapine for 24 h and then were fixed for BrdU staining. (**A**) Representatives of BrdU-labeled cells. (**B**) Statistical graph showing the ratio of BrdU^+^ cells. (**C–E**) Effect of fluoxetine and/or olanzapine on neuronal differentiation of neuron-cocultured NSCs. Neuron-cocultured NSCs were allowed to differentiate in the presence of 10 μM fluoxetine and/or 10 nM olanzapine for 4 days and then were fixed for β-III-Tubulin staining. (**C**) Representatives of β-III-Tubulin-labeled neurons. (**D**) Statistical graph showing the number of β-III-Tubulin^+^ neurons. (**E**) Statistical graph showing the percentage of β-III-Tubulin^+^ neurons. Scale bars = 50 μm. Data are mean ± SEM (*n*=3); *** *P*<0.001 versus Control group, # *P*<0.05 versus Fluoxetine group. Abbreviations: BrdU, bromodeoxyuridine.

To determine whether fluoxetine and/or olanzapine affect neurogenesis when NSCs are cocultured with neurons, we cocultured monolayer NSCs on coverslips with neurons, which had grown for 10 days *in vitro*, and meanwhile treated the cocultures with 10 μM fluoxetine, 10 nM olanzapine, or 10 μM fluoxetine plus 10 nM olanzapine. The effect of drug treatments for 24 h (10 μM BrdU was added at 22 h after drug treatments to label the dividing cells) on proliferation of neuron-cocultured NSCs is shown in Figure 3A,B (ratio of BrdU^+^ cells; Control, 25.0 ± 1.3%; olanzapine, 26.8 ± 1.4%; Fluoxetine, 40.0 ± 1.0%; Fluoxetine plus olanzapine, 40.4 ± 1.9%; F_(3,8)_ = 33.046, *P*<0.001). The neuron-cocultured NSCs, similar to the only cultured NSCs, also did not exhibit a markedly altered ratio of BrdU^+^ cells after olanzapine treatment. Unlike olanzapine, fluoxetine alone (*P*<0.001) and fluoxetine plus olanzapine (*P*<0.001) significantly promoted the proliferation of neuron-cocultured NSCs. However, fluoxetine plus olanzapine had no remarkable effect on cell proliferation compared with fluoxetine alone, which indicates that olanzapine has little synergism with fluoxetine in promoting the proliferation of neuron-cocultured NSCs. The effect of treatment with fluoxetine and/or olanzapine for 4 days on neuronal differentiation of neuron-cocultured NSCs is shown in Figure 3C–E (number of β-III-Tubulin^+^ neurons; Control, 10.1 ± 0.7; olanzapine, 10.8 ± 2.0; Fluoxetine, 14.9 ± 1.2; Fluoxetine plus olanzapine, 21.5 ± 1.3; F_(3,8)_ = 14.035, *P*=0.001; ratio of β-III-Tubulin^+^ neurons; Control, 14.8 ± 1.0%; olanzapine, 14.9 ± 2.3%; Fluoxetine, 15.0 ± 1.2%; Fluoxetine plus olanzapine, 22.1 ± 1.5%; F_(3,8)_ = 5.323, *P*=0.026). Fluoxetine alone elicited a nonsignificant increase in the number of neurons at day 4 after differentiation (Figure 3D). Furthermore, the percentage of neurons did not change after fluoxetine treatment (Figure 3E); note that fluoxetine does not appear to instruct NSCs to adopt a neuronal fate. Although olanzapine alone did not affect neuronal differentiation of neuron-cocultured NSCs, the presence of it substantially amplified the response to fluoxetine. The combination of olanzapine and fluoxetine significantly increased the number and ratio of neurons at day 4 after differentiation compared with fluoxetine alone (*P*<0.05). It follows then that olanzapine has synergism with fluoxetine in promoting neuronal differentiation of neuron-cocultured NSCs.

### Olanzapine has synergism with fluoxetine in promoting survival and neuronal fate choice of newborn hippocampal cells

**Figure 4:**
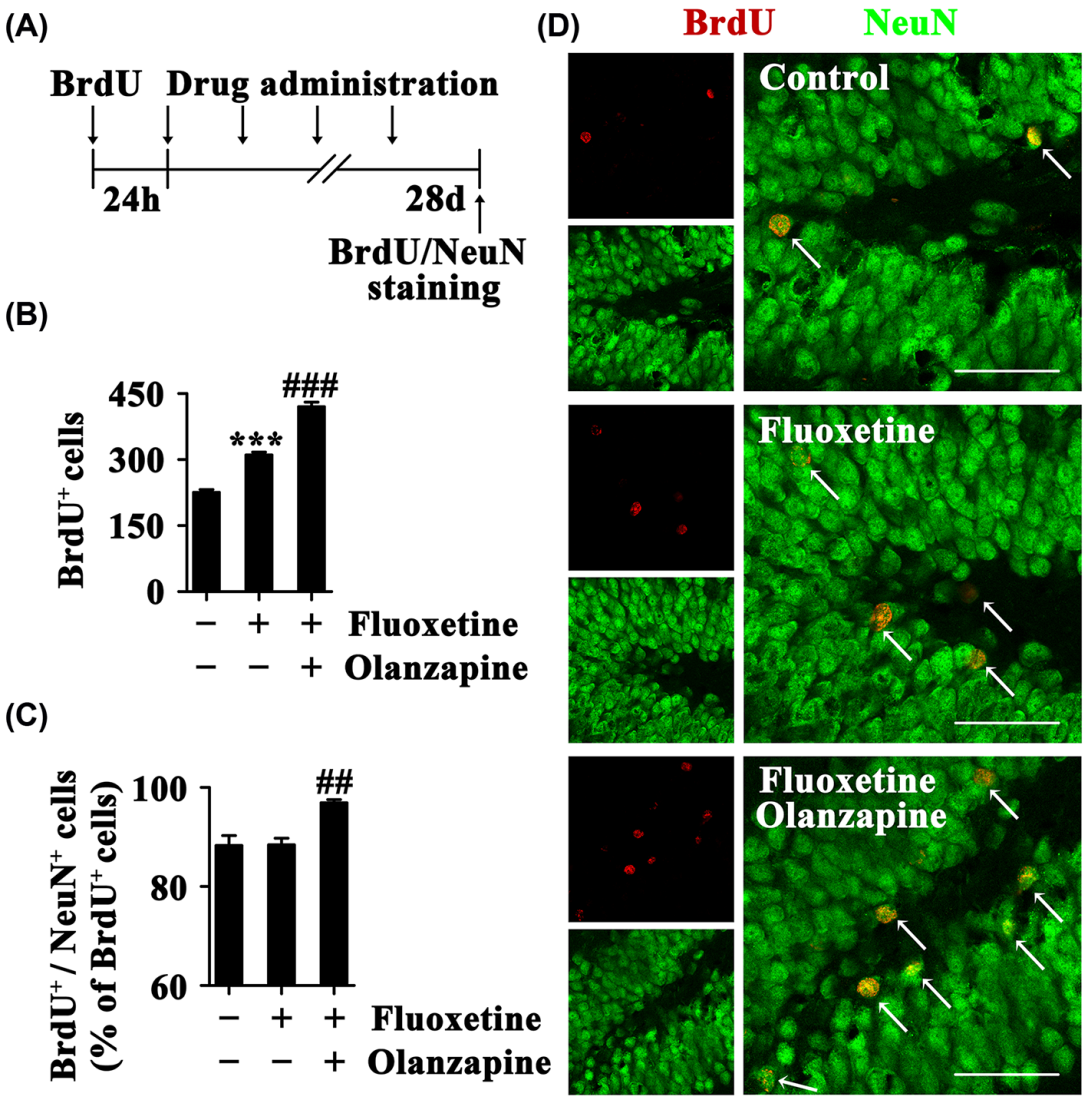
Olanzapine has synergism with fluoxetine in promoting survival and neuronal fate choice of newborn hippocampal cells (**A**) The schedule for drug and BrdU administration. Mice were administrated a single injection of BrdU; and 24 h later, the mice were treated with fluoxetine in the absence or presence of olanzapine once daily for 28 days. Mice were killed for BrdU and NeuN staining 24 h after the end of the drug treatment. (**B**) Bar graph showing the number of surviving BrdU^+^ cells in the ipsilateral dentate gyrus. (**C**) Bar graph showing the percentage of BrdU^+^/NeuN^+^ neurons among total BrdU^+^ cells in the ipsilateral hippocampus. (**D**) Images showing BrdU^+^/NeuN^+^ cells in the dentate gyrus. Scale bars = 50 μm. Data are mean ± SEM (n = 5); *** *P*<0.001 versus Control group; ## *P*<0.01, ### *P*<0.001 versus Fluoxetine group. Abbreviations: BrdU, bromodeoxyuridine.

To further investigate the synergistic effect of olanzapine and fluoxetine on promoting neuronal differentiation of NSCs in adult brains, we labeled dividing cells with BrdU (200 mg/kg, i.p.), treated mice with fluoxetine (10 mg/kg per day, qd, i.p.) in the absence or presence of olanzapine (3 mg/kg per day, qd, i.p.) for 28 days, and examined the number of surviving BrdU^+^ cells and BrdU^+^/NeuN^+^ neurons in the hippocampus (Figure 4A). The effects of drug administration on the number of surviving BrdU^+^ cells (Control, 225.6 ± 6.2; Fluoxetine, 310.8 ± 6.4; Fluoxetine plus olanzapine, 420.0 ± 10.7; F_(2,12)_ = 146.541, *P*<0.001) and the percentage of newborn neurons (Control, 88.3 ± 2.0%; Fluoxetine, 88.4 ± 1.3%; Fluoxetine plus olanzapine, 96.9 ± 0.6%; F_(2,12)_ = 12.059, *P*=0.001) are shown in Figure 4B–D. Treatment with fluoxetine resulted in an increase in the number of BrdU^+^ and BrdU^+^/NeuN^+^ cells but did not change the fraction of BrdU^+^/NeuN^+^ neurons among the total BrdU^+^ cells. The results indicate that fluoxetine does not alter neuronal fate choice of newborn hippocampal cells but promotes cell survival, which is the reason for increased number of newborn neurons. The presence of olanzapine enhanced the response to fluoxetine. The coadministration of olanzapine and fluoxetine significantly increased the number of surviving BrdU^+^ cells (*P*<0.001) and the percentage of newborn neurons (*P*<0.01) (Figure 4B–D). Therefore, olanzapine has synergism with fluoxetine in promoting the survival and neuronal fate choice of newborn hippocampal cells.

## Discussion

In the present study, we have examined whether olanzapine augments the effects of fluoxetine on NSCs proliferation and neuronal differentiation by cell culture *in vitro* and animal experiments. We demonstrated that although olanzapine alone does not affect proliferation and neuronal differentiation of NSCs, olanzapine add-on treatment produces a greater number and percentage of differentiated neurons from NSCs than fluoxetine alone. Combination of olanzapine and fluoxetine does not further enhance NSCs proliferation compared with fluoxetine alone.

In order to examine the synergism between olanzapine and fluoxetine in regulating neurogenesis, we planned to combine less-than-active concentration of olanzapine with fluoxetine. To determine the inactive concentration, olanzapine (10 nM, 100 nM, 1 μM, or 10 μM) was added to cultured NSCs. We did not detect any significant changes in NSCs proliferation and neuronal differentiation across all these olanzapine concentrations. Similar findings have also been reported for *in vitro* experiments that olanzapine (10 nM, 100 nM, 1 μM) has no detectable effects on cell proliferation and differentiation of subventricular zone (SVZ)-derived neural precursors [12]. However, *in vivo* studies report that chronic olanzapine treatment increases cell proliferation in subgranular zone of hippocampal DG and SVZ of lateral ventricles, two canonical sites of adult brains where continuously active neurogenesis exists [13–15]. The mixed results indicate that olanzapine is likely to regulate neurogenesis via indirect effect by surrounding cells.

Unlike olanzapine, fluoxetine regulates neurogenesis by acting both directly and indirectly on NSCs. Our data demonstrated that fluoxetine does not influence cell proliferation and inhibits neuronal differentiation of cultured NSCs. However, when NSCs are cocultured with neurons, fluoxetine promotes NSCs proliferation and does not affect neuronal fate commitment. Thus, we speculate that fluoxetine acts on neurons and increases the secretion of external proneurogenic factors such as BDNF [16]. Opposite bidirectional interactions between intrinsic programs and external factors cause no change by fluoxetine in neuronal differentiation of neuron-cocultured NSCs. Our *in-vivo* experiments showed that fluoxetine also does not alter neuronal fate choice of hippocampal newborn cells; of course, this is probably also the result of complex interactions between microenvironment and hippocampal NSCs. Although previous *in-vivo* studies have almost consistently concluded that fluoxetine promotes hippocampal cell proliferation and survival and does not affect neuronal fate [17–20], *in-vitro* studies on the regulation of neurogenesis by fluoxetine have been mixed. A study assessed the effects of different concentrations (0.001–20 μM) of fluoxetine on proliferation of cultured hippocampal NSCs and showed similar results to ours that 10 μM fluoxetine does not influence cell proliferation [21]. In addition, it reported that only 1 μM fluoxetine increases cell proliferation, but fluoxetine at this concentration has no effect on neuronal differentiation [21]. Another study has reported that 1 μM fluoxetine enhances proliferation of cultured hippocampal NSCs and encourages them to differentiate into neurons [22]. Additionally, 1 μM fluoxetine has also been shown to induce proliferation and inhibit neuronal differentiation of cultured hypothalamic NSCs [23]. It seems therefore that treatment with fluoxetine indisputably promotes cell proliferation at the right concentration but elicits a paradoxical result on neuronal differentiation of cultured NSCs.

Our data in different conditions have consistently showed that the combination of olanzapine and fluoxetine does not further enhance proliferation of NSCs compared with fluoxetine alone, but olanzapine add-on treatment produces a greater number and percentage of differentiated neurons from NSCs. Contrary to our results, the addition of quetiapine (another atypical antipsychotic) to fluoxetine increased hippocampal cell proliferation but had no effect on neuronal fate choice of newborn hippocampal cells in fluoxetine-resistant depressive rats [10]. To date, one study has reported fluoxetine plus olanzapine action on regulation of hippocampal neurogenesis [13]. In line with our data, the study did not prove a greater increase in cell proliferation for olanzapine add-on treatment when compared with fluoxetine alone. But unlike our results, the study showed that fluoxetine plus olanzapine did not increase cell survival and neuronal differentiation to a greater extent than fluoxetine alone. The combination treatment elicited a small but nonsignificant increase in the percent of NeuN and BrdU double-labeled newborn neurons (Control, 87.9%; Fluoxetine, 88.5%; Fluoxetine plus olanzapine, 93.3%). Olanzapine dose was 0.5 mg/kg per day in the study, and the higher dose of olanzapine might elicit significant effect.

The molecular mechanisms underlying the interaction of olanzapine and fluoxetine in regulating neurogenesis are not known. Fluoxetine increases extracellular level of 5-hydroxytryptamine (5-HT) by blocking the 5-HT transporter, and thereby indirectly activates 5-HT receptors. However, there are at least 14 5-HT receptor subtypes, and different subtypes have different or even opposing pharmacological effects [24,25]. Olanzapine exhibits antagonistic activity for 5-HT_2A/2C_, 5-HT_3_, 5-HT_6_, and dopamine D_1-5_ receptors, and has the strongest affinity for the 5-HT_2A_ receptor [26]. Therefore, low-dose olanzapine is most likely to produce the augmenting effect via saturating the 5-HT_2A_ receptor. Further studies are needed to explore the role of 5-HT_2A_ receptor in increased neurogenesis because of olanzapine in combination with fluoxetine.

## Data Availability

All available data can be obtained by contacting the corresponding author.

## Abbreviations

5-HT: 5-hydroxytryptamine
bFGF: basic fibroblast growth factor
BrdU: bromodeoxyuridine
DG: dentate gyrus
EGF: epidermal growth factor
FBS: fetal bovine serum
NSC: neural stem cell
PBS: phosphatebuffered solution
PFA: paraformaldehyde
SVZ: subventricular zone
